# Prediction of extracapsular extension of prostate cancer by MRI radiomic signature: a systematic review

**DOI:** 10.1186/s13244-024-01776-8

**Published:** 2024-08-26

**Authors:** Adalgisa Guerra, Helen Wang, Matthew R. Orton, Marianna Konidari, Nickolas K. Papanikolaou, Dow Mu Koh, Helena Donato, Filipe Caseiro Alves

**Affiliations:** 1 Department of Radiology, Hospital da Luz Lisbon, Lisboa, Portugal; 2 Royal Surrey County Hospital HSH Foundation Trust. Royal Marsden Hospital NHS Foundation Trust, London, England; 3 Royal Marsden Hospital NHS Foundation Trust, London, England; 4 Documentation and Scientific Information Service, Centro Hospitalar e Universitário de Coimbra, Coimbra, Portugal; 5 Faculty of Medicine, University of Coimbra, Coimbra, Portugal

**Keywords:** Systematic review, Radiomics, Machine learning, Prostate cancer, Extracapsular extension

## Abstract

**Abstract:**

The objective of this review is to survey radiomics signatures for detecting pathological extracapsular extension (pECE) on magnetic resonance imaging (MRI) in patients with prostate cancer (PCa) who underwent prostatectomy.

Scientific Literature databases were used to search studies published from January 2007 to October 2023.

All studies related to PCa MRI staging and using radiomics signatures to detect pECE after prostatectomy were included.

Systematic review was performed according to Preferred Reporting Items for Systematic Review and Meta-analyses (PRISMA). The risk of bias and certainty of the evidence was assessed using QUADAS-2 and the radiomics quality score.

From 1247 article titles screened, 16 reports were assessed for eligibility, and 11 studies were included in this systematic review. All used a retrospective study design and most of them used 3 T MRI. Only two studies were performed in more than one institution. The highest AUC of a model using only radiomics features was 0.85, for the test validation. The AUC for best model performance (radiomics associated with clinical/semantic features) varied from 0.72–0.92 and 0.69–0.89 for the training and validation group, respectively. Combined models performed better than radiomics signatures alone for detecting ECE. Most of the studies showed a low to medium risk of bias.

After thorough analysis, we found no strong evidence supporting the clinical use of radiomics signatures for identifying extracapsular extension (ECE) in pre-surgery PCa patients. Future studies should adopt prospective multicentre approaches using large public datasets and combined models for detecting ECE.

**Critical relevant statement:**

The use of radiomics algorithms, with clinical and AI integration, in predicting extracapsular extension, could lead to the development of more accurate predictive models, which could help improve surgical planning and lead to better outcomes for prostate cancer patients.

**Protocol of systematic review registration:**

PROSPERO CRD42021272088. Published: 10.1136/bmjopen-2021-052342.

**Key Points:**

- Radiomics can extract diagnostic features from MRI to enhance prostate cancer diagnosis performance.
- The combined models performed better than radiomics signatures alone for detecting extracapsular extension.
- Radiomics are not yet reliable for extracapsular detection in PCa patients.

**Graphical Abstract:**

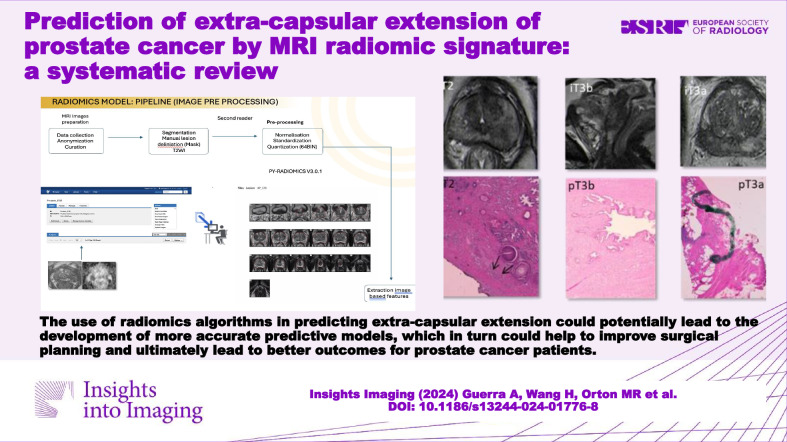

## Background

Prostate cancer (PCa) is a common cancer in Europe and all over the world, with around 6600 new cases diagnosed each year [1]. Radical prostatectomy is widely recognised as the standard surgical treatment for early-stage PCa. The detection of extracapsular extension (ECE) is fundamental for planning the surgical approach because it can lead to high rates of positive surgical margins, recurrence, and decreased survival [2–7]. Nomograms, such as D’Amico or CAPRA, are often used to predict the risk of advanced disease [8, 9]. Magnetic resonance imaging (MRI) has been shown to improve accuracy in predicting ECE, but there is high inter-reader variability related to the semantic features interpretation on MRI which is not consensual among the authors [10–12]. The high-quality MRI acquisition protocol and the high experience of the readers could help to improve the accuracy of MRI [10–12].

Radiomics can help extract different features from medical images using data-characterisation algorithms, improving diagnostic performance in PCa, as well as the reproducibility of the MRI examinations. Artificial intelligence (AI) and machine learning (ML) can help apply radiomics in everyday practice. However, clinically accepted and validated algorithms have not been established [13–15].

This systematic review aims to summarise evidence on using radiomics algorithms to predict pathological extracapsular extension (pECE) in PCa patients to aid surgical planning and improve outcomes.

## Methods

This systematic review follows the guidelines for Preferred Reporting Items for Systematic Reviews and Meta-analyses and the protocol was registered with PROSPERO (CRD42020215671) and published in BMJ Open [16].

### Eligibility criteria

This article reviewed manuscripts involving adult PCa patients who had a presurgical prostate biopsy indicating a Gleason score equal to or greater than 6 and underwent MRI before their surgery. Only studies using 1.5-T or 3-T MRI scanners and no prior treatment were included.

The primary outcome was pathologic local staging after surgery, with the goal of identifying imaging and clinical predictors of extracapsular extension on pathology specimens (pECE). The eligible studies were required to be retrospective or prospective cohort studies or randomised controlled trials that included prognostic factor analysis. Furthermore, these studies needed to have been published in peer-reviewed journals.

Studies were included if:

- Information regarding PCa MRI staging and pathological PCa staging was available in the published report.
- MRI images and radiomics signatures were used to detect pECE after prostatectomy.

Studies were excluded if:

- The AI/ML predictive models were built with another main predictive endpoint, such as localisation, segmentation, recurrence, prognosis, or lymph node metastasis, and characterisation of PCa without reference to the pathologic PCa staging endpoint. The paper was included in the analysis if the authors built different models with different endpoints but included the pathologic PCa staging endpoint.
- Studies with only MRI image characteristics, interpretative MRI semantic features, or combine feature for any signature without combining the radiomics feature extraction signature, were excluded.
- Cross-sectional studies, case series, case reports, case-control studies, systematic reviews, conference proceedings, and master’s or PhD theses were excluded.

### Search strategy

We conducted a comprehensive search across six electronic databases, namely CINAHL, EMBASE, CENTRAL (Cochrane Central Register of Controlled Trials via Wiley Online Library), PubMed, Web of Science Core Collection, and for grey literature, OpenGrey and Grey Literature Network Service. Furthermore, we manually searched through the reference lists of all included studies and previously published systematic reviews of MRI staging of PCa.

The search strategy was developed by a medical librarian with expertise in systematic reviews. The search terms were customised to the specific requirements of each database. Keywords (“Prostate neoplasm”, “Machine learning”, “Artificial intelligence” “Radiomics”, “Deep Learning”, “Staging” and “Magnetic Resonance Imaging”) or subject headings specific to each database (e.g., MeSH) were used along with Boolean operators ‘OR’ and ‘AND’ to combine the search terms effectively. The search strategy is detailed in the published protocol [16].

### Study selection

There were no restrictions applied, and only studies published in the English language were included. The search was conducted in each database from January 2007 to October 2023.

**Fig. 1:**
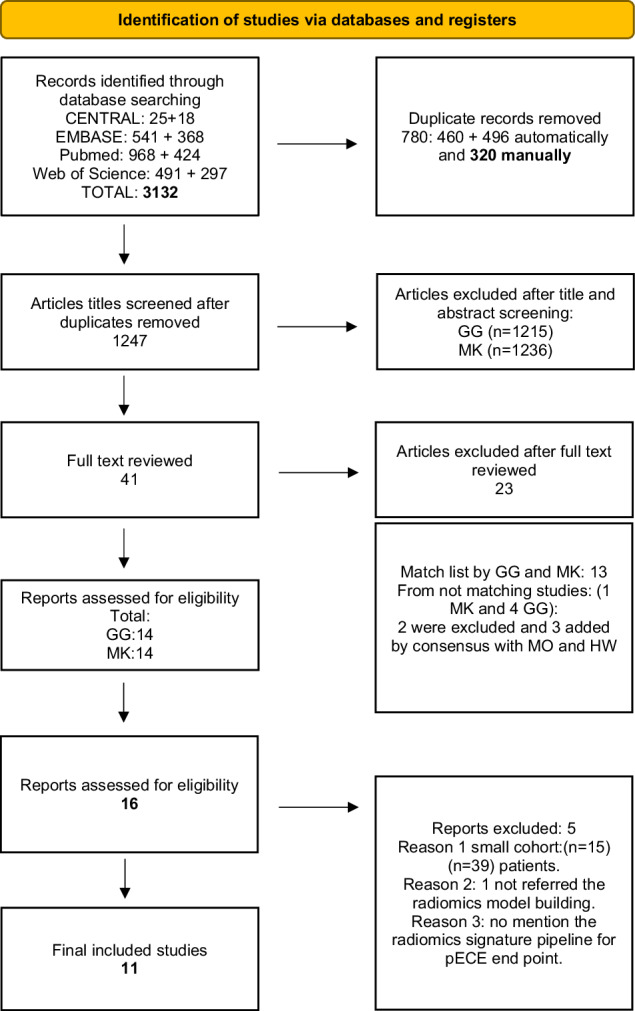
PRISMA flow diagram of the study selection

Following title/abstract screening, the full texts of potentially relevant studies were evaluated. In cases where a consensus was not reached between the two reviewers (A.G. and H.W.), a third reviewer (M.K.) was consulted. Additionally, the reference lists of the studies chosen for inclusion were examined for any other relevant studies. The data collection process is illustrated in Fig. 1.

### Data collection process

Data extraction: Two reviewers (A.G. and M.K.) independently extracted the following data from the included studies. In cases of disagreement between the two reviewers, a consensus was reached through discussion. If necessary, two additional expert reviewers (M.O. and H.W.) were consulted. The extracted data were broadly categorised into patient and study characteristics, radiologist details, type of feature extraction (agnostic if extracted by computation algorithms), semantic (interpreted by a radiologist), model characteristics, and predictive performance. Sensitivity, specificity, and area under the receiver operating characteristic curve (AUC) were extracted in the training and validation groups, with 95% confidence intervals where available. The radiomics and integrated models were compared, and the best predictive performances were registered.

### Risk of bias applicability

The risk of bias in individual studies was assessed by three reviewers (A.G., H.W., and M.K.). Since we included diverse types of studies, we used different tools to assess the risk of bias depending on the characteristics of the studies. Data from these studies were extracted, tabulated, and then reviewed for risk of bias and applicability using the Quality Assessment of Diagnosis Accuracy Studies version 2 (QUADAS-2) tool [17]. This tool covers four sources of bias: (1) patient selection, (2) index test, (3) standard domain, and (4) flow and timing bias. For each one, the risk of bias was assessed as high risk, unclear risk, or low risk, depending on the information offered by the study. The review authors used the signalling QUADAS-2 question information to judge the risk of bias. If all signalling questions for a domain were answered ‘yes’ then the risk of bias was considered ‘low’. If any signalling question was answered ‘no’ this flagged the potential for bias. The ‘unclear’ category was used only when insufficient data were reported to permit a judgement.

Because QUADAS-2 sometimes does not accommodate the niche of terminology encountered in AI studies, we also added a radiomics quality score (RQS) proposed by Lambin et al to this systematic review [17, 18]. Studies with a high risk of bias and low applicability were excluded. A narrative synthesis was conducted, acknowledging the risk of bias and the strength and consistency of significant associations.

### Synthesis of results

Due to differences in AI system applications, study designs, algorithms, patient cohorts, evaluation strategies, and performance metrics, narrative synthesis was chosen instead of meta-analysis. Meta-analysis could be not recommended for studies of diagnostic test accuracy that have significant differences in patient cohorts and test settings, as it would produce biased results.

## Results

### Studies characteristics (Table 1)

The eleven final included studies (corresponding to 0.009% from a total of 1247 screened papers), were published between 2019 and 2023, used a retrospective study design and were mainly (8) from China, two from Italy, and one from Norway. All the studies described a model based on radiomics extracted features, either alone [19–22], combined with clinical features [23–27], or in combination (integrated model with semantic interpretative features, plus agnostic radiomics features associated with clinical features to predict ECE in histopathological specimen analysis [28, 29]. All but three studies used a 3 T field strength and the total number of patients included in the models ranged between 62 to 284. Only two studies [23, 30]were performed in more than one institution. The lesion segmentation and feature interpretation were undertaken by more than one radiologist, and the inter-agreement ratio was evaluated in all studies, except Losnegård et al [28]. A recent study [29] compared three individual models: radiomics, clinical and the assessment of ECE on MRI done by four radiologists: semantic model) and compared them to a combined model with all relevant features from the three models. Three studies also referred to other endpoints, such as the positivity of surgical margins [24, 26], lymph node metastases and tumour aggressivity [19, 26]. One study also built a radiomics model to predict PCa prognostic biological biomarkers [24].

**Table 1:** General characteristics of the studies

Author	Year	Country	Semantic features^a^	ROI (radiomics features)	Clinical features	Combine models	Type of study	Total patients	Training group	Validation group	Number of institutions	Readers	External validation	Field strength
Ma et al [20]	2019	China	No	Manual extracapsular tissue	No	No	Retrospective	119	74	45 (internal validation)	1	2	No	3 T
Ma et al [21]	2019	China	No	Capsule and extracapsular tissue	No	No	Retrospective	210	143	67 (internal validation)	1	3	No	3 T
Losnegard et al [28]	2020	Norway	Yes	8:2 (lesion) and 6 (whole prostate)	MSKCC nomogram	Yes	Retrospective	228	Not available	Not available	1	1	No	1.5 T Endo coil
Xu et al [25]	2020	China	No	Lesion on DWI	tPSA, fPSA, PSAf/tPSA, GS	Yes	Retrospective	95	82	33 (internal validation)	1	2	No	3 T
Bai H. et al [23]	2021	China	No	Peri-tumoural (automatically) & manual intratumoural	tPSA, GS (prostate biopsy)	Yes	Retrospective	284	158	68 (internal validation) 58 (external validation)	2	2	Yes	3 T
Cuocolo R. et al [22]	2021	Italy	No	Manual on lesion	No	No	Retrospective	193	104 (site 1)	43 (site 2), 46 (site 3)	3	3	Yes	1.5 & 3 T
Damascelli [19]	2021	Italy	No	Manual on lesion	No	No	Retrospective	62	Not available	No	1	2	No	1.5 T Endo coil
He et al [26]	2021	China	No	Lesion on ADC and T2WI	Age, tPSA, fPSA, (fPSA/tPSA), GS biopsy, % positive biopsy cores	Yes	Retrospective	273	192	81 (internal validation)	1	3	No	3 T
Fan et al [24]	2022	China	No	Manual on lesion on DWI	Age, PSA, WBC, RBC, haemoglobin, lymphocyte, platelet, albumin, ALP, PLR, fibrinogen	Yes	Retrospective	232	185	47 (internal validation)	1	2	No	3 T
Yang Liu [27]	2023	China	No	Lesion on T2WI	Age, Gleason score P504s	Yes	Retrospective	86	no	no	1	2	No	3 T
Liqin Yang et al [29]	2023	China	Yes	Lesion on T2, ADC and DWI	Age PSA, lesion localisation GS biopsy	Yes	Retrospective	392	274	118	1	4	No	3 T

*tPSA* prostate-specific antigen total, *fPSA* prostate-specific antigen free, *GS* Gleason score, *WBC* white blood cell, *RBC* red blood cell, *ALP* alpha fetoprotein, *PLR* platelet-to-lymphocyte ratio, *MSKCC* Memorial Sloan Kettering Cancer Center, *1.5* *T* tesla, *3* *T* tesla, *T2WI* weighted image, *DWI* diffusion-weighted imaging

^a^ Interpreted by radiologist

### Radiomics characteristics (Table 2 and Table-S1)

#### Model performance

All studies reviewed focus on developing radiomics models based on agnostic features extracted from T2WI (T2-weighted imaging) and ADC (apparent diffusion coefficient) of the manual segmented tumoural region. DCE images were also used in four publications [20, 21, 24, 28]. They compared different signatures composed by IFs (imaging features) extracted from T2WI and ADC maps independently and from the two modalities, and they tested them for prediction presence vs. absence of pECE.

**Table 2:** Radiomics characteristics

Author	Vendors	Input sequences	Vendor harmonisation	Image processing	Radiomics extraction	Algorithm	Evaluation strategy	SN	SP	AUC	95% CI	SN	SP	AUC	95% CI	AUC (radiomics signature alone)	95% CI
								Training group^a^				Validation group^a^					
Bai H. et al [23]	2/Siemens. 2/GE. 2/United Imaging Health care	T2WI, ADC	*Z*-score normalisation	Yes	Pyradiomics	10-folds CV logistic regression with LASSO	Randomly internal validation (30%) external validation.	0.783	0.723	0.723	Not reported	0.68	0.667	0.692	Not reported	0.7	Not reported
Cuocolo R. et al [22]	2/Philips. 1/Siemens	T2WI, ADC	*Z*-score normalisation	Yes	Pyradiomics	10-fold cross-validation SVM	External validation	0.83	0.83	0.83	Not reported	0.79 and 0.74	0.80 and 0.74	0.80 and 0.73	Not reported	n/a	Not reported
Damascelli [19]	1/Philips	T2WI, ADC	Not required	Yes	3D slicer v 4.10.2	unsupervised hierarchical clustering,	No validation	0.88	0.89	0.88 (accuracy)	0.82–0.9		n/a	n/a		0.88 (training)	0.82–0.91
Fan et al [24]	3/GE. 2/Siemens	T2, DWI, DCE	Not required	No	Pyradiomics	random Forest	Internal validation	0.85	0.88	0.95		0.71	0.9	0.85		0.93 (training) 0.84 (validation)	
He et al [26]	1/Siemens	T2WI, ADC	Not required	Yes	Pyradiomics	10-folds CV logistic regression with LASSO	Internal validation	Not reported	Not reported	Not reported		0.727	0.688	0.728	0.821	0.625	0.724
Losnegard [28]	1/Siemens	T2WI, T1WI, DWI, DCE	Not required	Yes	Matlab	10-folds CV random forest	No validation	0.79	0.57	0.75	SD 0.01	n/a	n/a	n/a		0.75	SD 0.06
Ma et al [20]	1/Philips. 1/GE	T2, DWI, DCE	Collewet normalisation	No	Matlab	10-folds CV logistic regression with LASSO	Internal validation	0.906	Not reported	Not reported	0.847, 0.948	0.821	Not reported	Not reported		0.821	0.726, 0.894
Ma et al [21]	1/Philips. 1/GE	T2, DWI, DCE	No	No	Matlab	10-folds CV logistic regression with LASSO	Internal validation	0.768	0.919	0.902	0.840, 0.945	0.75	0.914	0.883	95% CI: 0.781, 0.949	0.883	0.781, 0.949
Xu et al [25]	2/Siemens. 2/GE. 2/United Imaging Health care	T2WI, ADC	Not required	Yes	Pyradiomics	10-folds CV logistic regression with LASSO	Randomly internal validation (30%), external validation.	0.829	0.894	0.91	0.861–0.978	0.714	0.895	0.865	0.738– 0.992	0.865	0.738– 0.992
Liqin Yang et al [29]	1 Siemens /Skyra)	T2WI, ADC, DWI	Not required	Yes harmonisation	Pyradiomics /FEAture explorer: FAEv0.4.0	LASSO	Randomly internal validation	^b^0.817	^b^0.818	^b^0.897	0.861–0.934	^b^0.90	^b^0.73	^b^0.89	0.861–0.934 0.837–0.950	0.882 training 0.835 validation	0.844–0.921 0.763–0.908
Yang Liu [27]	1 GE discovery	T2WI,	Not required	No	Pyradiomics. Slicer radiomics	LASSO	No validation	0.952	0.523	0.763	0.662–0.864	n/a	n/a	n/a	0.861–0.934 0.837–0.950	0.739	0.631– 0.846

*SN* sensitivity, *SP* specificity, *AUC* area under the curve, *GE* general electrics, *CV* cross-validation, *SVM* support vector machine, *LASSO* regression analysis method

^a^ Best results model

^b^ In case of combine models (radiomics + clinical + Mehralivand grade)

For each MRI sequence, shape features (size and sphericity) and texture features (GLCM, GLRLM, GLSZM, NGLDM) were the most common features with discriminative importance and in the majority of cases were for those extracted from T2WI. The exception was Fan et al [24], where the most relevant feature was extracted from DCE. The radiomics features derived from histograms were not so relevant as the features previously mentioned. The coefficients for the calculation of the selected radiomics features were different between the studies and the authors did not find a common stable radiomics feature which could be the dominant impact factor for pECE across them. The image processing and feature selection methods were very heterogenous between the studies. Matlab, original Pyradiomics and Laplacian of Gaussian (LoG) and wavelet-filters were used for images extraction. Most researchers compared radiomics with clinical and combined models (radiomics + clinical features). In these cases, the combined models achieved the best performance (AUC: 0.92) [25], 0.72 [26], 0.95 [24], 0.72 [23], 0.76 [27] and 0.89 [29].

The highest AUC of a model using only radiomics features (tumoural region) to predict ECE was 0.93 for the training group and 0.85, for the test validation [24]. This was followed by Xu et al [25] (AUC 0.91), Yang et al [29] (AUC 0.86) and Cuocolo et al [22] (AUC 0.83 and 0.80/0.73, in training set and two external validation sets, respectively).

Ma et al [20, 21] built a radiomics signature in the peri-tumoural region (capsule and periprostatic fat) and compared it with the radiologist’s interpretation. Pairwise comparisons showed that the radiomics signature was more accurate than the radiologist’s interpretation. The accuracies (90% and 88%, respectively, in the training and validation groups) were much higher than that achieved directly by the radiologists (AUCs 0.685–0.755 in the training cohort and 0.600–0.697 in the validation cohort). This study is aligned with Yang et al study [29], where the radiomics signature is superior to radiologist interpretation (AUC 0.88 and 0.835, training and validations groups vs. AUC 0.746 and 0.774 training and validations groups) respectively.

Bai et al compare intra and peri-tumoural (PT) single radiomics signatures and achieve the best predictive value AUC: 0.70., in the PT region extracted from the ADC map. In this study the PT were automatically derived through 3D dilatation and was extracted from ADC map.

Only two authors [28, 29] built a combined model with clinical and semantic interpretative MRI features using Mehralivand’s proposed EPE-grade criteria [31]. They compared the radiologist’s interpretation and the Memorial Sloan Kettering Cancer Center (MSKCC) nomogram with the radiomics signature and the combined models. The AUC of the radiologist’s interpretative model was similar in both studies in the training group (AUC of 0.74) [28, 29] and 0.77 in the validation group in the study of Yang et al [29]. In relation to radiomics model, the AUC was in both studies, AUC 0.75 [28] and 0.88 [29], respectively.

The combination of radiomics, radiology plus clinical interpretation performed statistically better (AUC 0.89; *p* < 0.05) than clinical model (AUC 0.74) and semantic model (AUC 0.77) but not statistically significantly (*p*-value 0.167) than the radiomics alone (AUC 0.835) [29].

#### Risk of bias assessment (Table 3)

The review authors used the QUADAS-2 and RQS methods to judge the risk of bias.

**Table 3:** Risk of bias: QUADAS-2 and radiomics quality scores

Risk of bias	Xu [25]	MA [20]	MA [21]	Losnegard [28]	FAN [24]	He [26]	Damascelli [19]	Cuocolo [22]	Bai [23]	Liqin Yang [29]	Yang Liu [27]
QUADAS-2											
Patient selection	Low	Low	Low	Unclear	Low	Low	High	Unclear	Low	Low	Unclear
Index test (MRI)	Low	Low	Low	High	Low	Unclear	Low	Low	Low	Low	Unclear
Reference standard (pECE)	Low	Low	Low	Low	Low	Low	Low	Low	Low	Low	Unclear
Flow and timing	Low	Low	Low	Low	Unclear	Low	Unclear	Low	Unclear	Low	Low
Global	Low	Low	Low	High	Unclear	Unclear	High	Unclear	Unclear	Low	Unclear
Radiomics quality score (RQS)											
Image protocol quality	2	2	2	2	2	2	2	2	2	2	1
Multiple segmentations	1	1	1	1	1	1	1	1	1	1	1
Phantom study on all scanners	0	0	0	0	0	0	0	0	0	0	0
Imaging at multiple time points	0	0	0	0	0	0	0	0	0	0	0
Feature reduction or adjustment for multiple testing	3	3	3	3	3	3	3	3	3	3	3
Multivariable analysis with non-radiomics features	1	0	0	1	1	1	0	0	1	1	1
Detect and discuss biological correlates	0	0	0	0	0	0	0	0	0	0	0
Cut-off analyses	1	1	1	1	1	1	1	1	1	1	1
Discrimination statistics	2	2	2	2	2	2	2	2	2	2	1
Calibration statistics	2	2	2	0	2	2	2	2	1	2	1
Prospective study registered in a trial database	0	0	0	0	0	0	0	0	0	0	0
Validation	2	2	2	−5	2	2	−5	4	3	2	−5
Comparison to ‘gold standard’ (pECE)	2	2	2	2	2	2	2	2	2	2	2
Potential clinical utility	2	2	2	2	2	2	2	2	2	2	2
Cost-effectiveness analysis	0	0	0	0	0	0	0	0	0	0	0
Open science and data	0	0	0	0	0	0	0	1	0	0	0
**Total**	**18**	**17**	**17**	**9**	**18**	**18**	**10**	**20**	**16**	**18**	**8**

*pECE* extracapsular extension in pathology, *MRI* magnetic resonance imaging

**QUADAS-2**

Patient selection: Only Damascelli et al [19] study was deemed to have a high risk of bias as the case selection process was unclear due to insufficient description.

The cases of Cuocolo et al [22], Losnegård et al [28] and Yang Liu [27] were considered unclear because they did not mention whether they excluded patients with any type of treatment before radical prostatectomy.

Index test (MRI Images): All patients underwent adequate and identical institutional MRI protocol. Manual segmentation of the lesions was reproducible in all the studies except one [28], in which only one radiologist undertook the lesions’ segmentation, and one study [26] did not exclude poor-quality images.

Reference standard: The risk of bias for the reference standard (presence of ECE in the specimen) was low in all of the included studies.

Flow and timing: Except for four studies [19, 23, 24, 27], which did not mention the time between MRI and prostatectomy, all the included studies were consistent in using appropriate reference standards, for patients and maintaining appropriate intervals between MRI and obtaining histopathology.

### Radiomics quality score

Cuocolo et al [22] study had the maximum RQS of 20 points, and Damascelli et al [19], Losnegård et al [28] and Yang Liu had the lowest scores of 10, 9 and 8 points, respectively. The main reason for this was the absence of model validation. No study was prospective or presented a phantom study on all scanners or imaging analyses at multiple time points. Only one study demonstrated open science data [22]. A cost-effectiveness analysis or biological correlation was not performed in all the studies.

## Discussion

This systematic review found ten studies that aimed to predict pECE in PCa patients using radiomics signatures. Most of these studies had limited sample sizes and used data from a single centre, and four used a single MR scanner, which restricts the generalisability of their models. All the models utilised textural feature extraction, but the most significant textural features varied among the studies. The majority of the significant features were extracted from T2WI.

The Damascelli et al study being referred to here has a high risk of bias, did not perform external validation, and its patient sample size of only 62 patients was not considered adequate for robust conclusions [19]. Cuocolo et al achieved an accuracy of 83% in the training group using only ROIs of intraprostatic lesions to predict pECE [22].

Ma et al did two complementary studies comparing the radiomics model built from the first study [20] with a semantic interpretative model MRI EPE grade, in the second study [21]. They found that the radiomics model achieved higher accuracy compared to the performance of radiologists as described in the results. The low accuracy of the radiologists may be due to the difficulty in determining macroscopic ECE involvement using limited visual interpretive findings. The radiomics model has a low risk of bias, as assessed by the QUADAS tool and RQS scale, but its MATLAB feature generation approach is not open-source and uses non-standard techniques, making it difficult to replicate. The study performed an internal training and validation split of 2:1 but, did not specify which dataset was used for feature selection, which may have affected the results. The model has not been independently evaluated at another institution, and further studies are needed to validate its performance. Nevertheless, this study suggests that it is possible to use peri-tumoural regions to create radiomics signatures for predicting ECE [21].

In relation to the remaining studies [23–27], clinical features were used to construct combined models in addition to the radiomics features. The dominant clinical predictors were the serum PSA and Gleason Score (GS) of the biopsy. They reported better results in combined models with clinical and radiomics variables compared with models using radiomics features alone, with values that varied between 72% and 95% and with moderate risk of bias in the QUADAS-2 evaluation and RQS between 16 and 18 points. From these, Fan et al [24] study had the best accuracy, and the textural extraction features were derived from the ROI drawn on DWI. However, it is a single institution study using two different scanners with an internal validation dataset.

Two studies [28, 29], created a combined model that associated semantic features from the radiologist’s interpretation with the radiomics model and clinical features. In the study [28] the AUC of radiomics model is almost the same than sematic interpretative model (AUC 0.75 vs. AUC 0.74, respectively). However, the model was executed in a limited way, without a validation group, and the risk of bias was very high (high in QUADAS and only 9 in RQS). In the other [29], more recent research, the authors proved that a combined model achieved the best AUC in the validation group compared to the other models. However, this study should be conducted to utilise external cohorts (form different institutions) to validate the robustness of radiomics and combined models to detect ECE.

While all the studies used one or more feature selection strategies, in order to reduce the overfitting. In addition, the use of different feature sets in different studies led to a lack of consistency in the features present in the final models, as previously mentioned, precluding any attempt to synergistically analyse the relevant radiomics features for predicting pECE across studies.

Future radiomics studies must ensure high-quality data collection and standardisation of radiomics features across different institutions and imaging protocols. The IBSI (Image Biomarker Standardisation Initiative) seeks to provide image biomarker nomenclature and definitions, benchmark datasets, and benchmark values to verify image processing and image biomarker calculations, as well as reporting guidelines, for high-throughput image analysis.

By addressing these concerns, future radiomics studies can enhance the reliability and clinical utility of radiomics signatures in detecting ECE in patients with PCa before surgery.

This review had certain limitations. Firstly, although our search strategy was comprehensive, there could be studies that were published between our search period and the publication of this review. Secondly, this systematic review only focused on radiomics signatures and did not analyse other AI methods, semantic interpretative scores or nomograms to detect ECE.

Finally, this review would have benefited from a quantitative synthesis or metanalysis of the analysed articles, but unfortunately was not possible as key statistical data and the dominant features were not reported for this small sample of studies.

## Conclusion

Non-imaging biomarkers such as PSA and GS have shown promise in predicting ECE in PCa. When combined with MRI data, Radiomics signatures could enhance accuracy in predicting ECE. However, current evidence lacks robustness to support the clinical use of radiomics signatures for ECE detection pre-surgery. Future radiomics studies need prospective testing in multicentre settings with large datasets, including external validation cohorts, to enhance reliability and clinical utility in detecting ECE.

### Supplementary information

ELECTRONIC SUPPLEMENTARY MATERIAL

## Data Availability

This is a systematic review based on previously published data, therefore no participant recruitment takes place.

## Abbreviations

AI: Artificial intelligence
ECE: Extracapsular extension
ML: Machine learning
PCa: Prostate cancer
pECE: Extracapsular extension in the prostatectomy specimen
QUADAS-2: Quality Assessment of Diagnosis Accuracy Studies version 2
RQS: Radiomics quality score
