# Supplementary material for: Prediction of extracapsular extension of prostate cancer by MRI radiomic signature: a systematic review
Source: Insights Imaging. 2024 Aug 26;15:217. doi: 10.1186/s13244-024-01776-8 (PMC11347513; doi:10.1186/s13244-024-01776-8)
Supplement: Supplementary file 1 — ELECTRONIC SUPPLEMENTARY MATERIAL [file 13244_2024_1776_MOESM1_ESM.pdf]

Prediction of extra-capsular extension of prostate cancer by MRI radiomic signature: a systematic review

ELECTRONIC SUPPLEMENTARY MATERIAL

Insights Imaging (2024) Guerra A, Wang H, Orton MR.

Table S1: Supplementary information

**Author**	ROI	Image processing	Radiomics Extraction	Discriminative features with high importance	Feature Selection	Algorithm
Bai H. et al [23]	Manual delineation of ITR of the largest lesion on axial images of the T2WI, ADC by radiologist 1, check and corrected by radiologist 2. Annular PTR automatically derived through 3D dilation of half of the tumor length and limited to 3–12 mm. The rectum, urethra, and bladder are manually removed.	Rigid registration with trilinear interpolation of ADC map to T2WI, ROI transferred. All scans were then interpolated to 0.5 mm in-plane resolution and 2 mm slice thickness.	Pyradiomics, no mention of parameters 18 first-order statistics features, 14 shape-based, 24 GLCM, 16 GLRLM, 16 GLSZM, 5 NGTDM, and 14 GLDM. Seven separate filters, including gradient, wavelet, logarithm, square, square root, exponential, and LBP3D<br>Total: 1595 features	ADC:<br>Shape_Flatness<br>Gradient_GLSZM_ SALGLE<br>Wavelet-LLH_GLRLM_ LRHGLE<br>Wavelet-LHL_GLCM _InverseVariance<br>Wavelet-LHL_GLSZM_ SAE<br>Wavelet-LHH_GLCM _ClusterShade<br>Wavelet-HLL_firstorder_Kurtosis<br>Wavelet-HLL_GLCM _MaximumProbability<br>Wavelet-HLL_ NGTDM _Strength<br>Wavelet-HLH_firstorder_Uniformity<br>Wavelet-HHL_firstorder_Mean<br>Wavelet-HHL_GLSZM_ SALGLE	ICC > 0.75 on 50 randomly selected cases repeat segmented by reader 1 and 3. Near-zero variance, percentage of distinct values out of the number of total samples below 10%. Pearson's correlation < 0.9. mRMR method was employed to finalize the top-ranked 22 features	10-folds CV Logistic Regression with LASSO:<br>Univariate for radiomics of ITR, PTR and ITR + PTR extracted features models and combine models (clinical + radiomics) on T2WI, ADC, and both. multivariable logistic regression for radiomics and clinical features.
Cuocolo R. et al[22]	Reader 1: manual segmentation of the entire index lesion volume on both T2w and ADC images. Reader 2 and 3 independently annotated a subset of 30 randomly selected patients from the site 1 training set.	voxel resampling to 1 × 1 × 1 mm, whole-image gray level z-score normalization, scaling by a 100 factor and array shift (by 300), followed by discretization with a fixed bin width (= 5).	Pyradiomics, original and filtered images were used to calculate 3D shape, first order, and texture features. In detail, the Laplacian of Gaussian filtering with multiple sigma values (= 1, 2, 3, 4, 5) and wavelet decomposition with all combinations of high- and low-pass filters in the x-, y-, and z-axes<br><br>2436 features	T2WI:<br>GLSZM_ SZNN<br>LoG-sigma-2-0-mm-3D_GLCM_ClusterShade<br>LoG-sigma-4-0-mm-3D_GLSZM_ SZNN<br>LoG-sigma-4-0-mm-3D_GLDM_ SDE<br>Wavelet-LHH_ GLCM _Correlation<br>Wavelet-HHL_GLCM_ IMC2<br>Wavelet-HHL_ GLDM_LDLGLE<br><br>ADC:_<br>GLDM_ LDLGLE<br>LoG-sigma-2-0-mm-3D_GLCM_IDN<br>LoG-sigma-2-0-mm-3D_GLDM_LDLGLE<br>LoG-sigma-4-0-mm 3D_firstorder_Maximum<br>Wavelet-LHL_GLSZM_GLV<br>Wavelet-HLH_ GLRLM_ LRE<br>Wavelet-HHH_ GLRLM_ LRLGLE	ICC ≥ 0.75 between 3 readers, a variance filter (threshold = 0.1), pairwise correlation (threshold > 0.8)	Training set classes were balanced using the Synthetic Minority Oversampling Technique. A stratified 10-fold cross-validation SVM was used for model tuning prior to final training on the entire site 1 dataset. The final model was an SVM classifier with a radial basis function kernel, C = 3 and gamma = 0.01;
Damascelli et al[19]	2 readers semi-automated segmentation with region growing on T2w and ADC. Largest lesion selected; VOI greater	VOIs were resampled to isotropic voxel spacing, using an up sampling scheme based on slice thickness. Collewet normalisation, intensities were	Radiomics module of 3D slicer v. 4.10.2. 93 IFs) was extracted from both T2w images and ADC, without filters. (14 morphological features (M), 18 first-	T2WI:<br>Sphericity, GLSZM_SAHGLE, GLSZM_LAHGLE, GLRLM_SRHGLE<br><br>ADC:<br>Elongation, Flatness,	First 10 stable features from nonparametric repeated measurement Friedman test were used to calculate a stability rank per	Features presenting extreme values were log2 transformed, replacing zero values with the smallest positive value of the radiomic phenotype. Z-score

	0.7 cc in ADC were included	discretized to a fixed number of 64 bins.	order statistical features from intensity histogram, (FOS), including mean ADC value, and 61 textural features from analysis of GLCM, GLSZM, GLRLM, NGTDM.<br><br>186 features	Inverse variance, Cluster shade, Busyness, Mean	feature from Independent segmentations performed by two readers. Clusters of highly correlated features (r > 0.8) were reduced to a single representative imaging feature, using the one presenting the highest inter-subject's range, calculated by Coefficient of Variation.	normalisation. 10 random subsets with undersampling. SVM with radial basis kernel.
Fan et al[24]	reader 1: manually delineation on axial-DWI, copied to T2 and DCE, with further modifications if required. Reader 2: revision, if in objection to the results, the results would be discussed and resegmented until a consensus was reached.	not reported	Pyradiomics. Original image and 8 filters (Laplacian of Gaussian (LoG), wavelet, square, square root,logarithm, exponential, gradient, andlocal binary pattern (LBP).) to extract first-order, shape, and texture features: GLCM, GLDM, GLSZM, GLRLM, NGTDM.<br><br>5343 features	DCE_Wavelet HLL GLRLM_SRHGE T2_Wavelet_LHL_GLCM_IDN	Z-score normalisation. Top 20 features from for ECE, PNI, and SM groups and 15 features for Ki67 and S100 groups in the training cohort using mRMR. Recursive feature elimination (RFE) based on random forest (RF) was applied to find the best feature combination.	SVM with polynomial kernel, K-nearest neighbor (KNN), random forest, decision tree, logistic regression, and XGBOOST. Radiomics only, clinical only and combined model.
He et al[26]	manually delineated slice by slice on both the T2WI and ADC sequences. Radiologist 1 performed a series of delineations at two different times, and radiologist 2 performed the delineation only once.	quantized to 32 bins	Pyradiomis, original and wavelet filter, first-order, shape, and texture features: GLCM, GLDM, GLSZM, GLRLM, NGTDM. .<br><br>851 features	ADC:<br>Shape_MeshVolume<br>Firstorder_10Percentile<br>GLSZM_SZN<br>Wavelet-LHH_GLSZM_SZN<br>Wavelet-HLH_GLDM_DNN<br>Wavelet-LLL_GLSZM_LGLZE	Interobserver and Intraobserver ICC ≥ 0.75. Variance close to 0. A Spearman correlation < 0.9. Z-score normalization, mRMR.	Logistic Regression with LASSO and 10-folds cv. Radiomics only model and radiomics + clinical features model.
Losnegard[28]	1 reader manual segmentation, 1 automatic atlas-based segmentation using the Advanced Normalization Tools segmentation. segmenting volumes were extracted from the dataset and prepared for feature extraction, two from the lesions and six from the whole prostate. Using morphological dilation, we added 2mm margins around the capsule to both the manually delineated and the auto-segmented prostates, and the resulting volumes were denoted "Prostate, dilated." Further, to extract the volume of the prostate capsule, voxels were	Lloyd-Max quantization	Matlab,<br><br>254 radiomics features	T2:<br>GLCM: Energy, Homogeneity, Entropy, Dissimilarity, Contrast<br>GLRLM: GVL, LRE, SRE, RLN, RP<br>GLSZM_LZLGE<br><br>ADC:<br>GLSZM_LZLGE		Random Forest with 10-fold cv with varying the number of selected features from 1 to 30, with 100 repeats.

	subtracted from the dilated prostate to no further than 5 mm inside the capsule, and the resulting volume was denoted “Prostate border.”					
Ma et al[20]	2 readers: Reader 1 manual delineated along the prostate capsule and within the surrounding tissues at each side of the prostate. Specifically, the prostatic contour itself and approximate 1 mm into the prostatic tissue were suggested to be included to extract the whole subtle structural features of the capsule as much as possible. On the other hand, surrounding tissues (i.e., neurovascular bundle, extra-prostatic mass, and periprostatic fat) suspected to be malignant and invasive growth of the PCa should also be included in the ROIs. Repeat within the same week. Reader 2, randomly segment 50	not reported	Matlab, 1619 radiomics features that based on three main categories (i.e., 15 global features, 4 non-texture features, and 1600 texture features)	GLCM: Entropy, homogeneity, variance<br>GLRLM : LRHGLE,LRLGLE<br>GLSZM: HGLZE, LZE, LZHGE, LZLGE, SZHGE,<br>NGTDM: busyness, contrast<br>Non-textural: size	Kendall correlation coefficient > 0.2	Logistic Regression with LASSO and 10-folds cv. The final radiomics signature was created based on a linear combination of the optimal features that were weighted by their respective coefficients.
Ma et al[21]	reader 1: manual delineation of ROI included the adjacent prostate capsule and surrounding tissues (eg, the periprostatic fat and extra-prostatic mass) on T2WI transverse sections. Reader 2, randomly selected 50 lesions	not reported	Matlab, 1619 radiomics features that based on three main categories: 15 global features, 4 nontexture features, 23 GLCM, 20 GLRLM, 40 GLSZM, 71 NGTDM	GLCM: Energy, autocorrelation, dissimilarity, sumaverage<br>GLRLM: GLN, LRE<br>GLSZM: SZLGE, GLN, SZN, SAE, SZHGE, HGLZE<br>NGTDM: busyness	Kendall correlation coefficent > 0.2, Logistic Regression with LASSO and 10-folds cv.	Linear Regression of the final selected features.
Xu et al [25]	Axial T2WI, DWI, ADC, and DCE images were displayed simultaneously and segmented, respectively, and high b-value DWI images were chosen for segmentation.<br>Reader 1: manual segmentation;<br>Reader 2: manual segmentation of half of the lesions (115/2), as well as revision of segmentation. The readers negotiated to reach an agreement for controversial cases.	spacing standardization to 1mm in all directions using sitkBSpline package in python SimpleITK.<br>Quantisation not reported.	Pyradiomics: original, wavelet and Laplace of Gaussian filter; first-order, shape, and texture features: GLCM, GLDM, GLSZM, GLRLM, NGTDM.<br><br>4580 features	T2:<br>LoG-sigma-5mm_GLSZM_GLN<br>DWI:<br>Log-sigma-4mm-GLRLM-SRLGLE<br>LoG-sigma-4mm_GLRLM_ LGLRE<br>LoGsigma3mm_GLRLM_ HGLRE<br>ADC:<br>Wavelet-HLH-GLSZM-GLNN<br>LoGsigma5mm_GLSZM_LGLZE<br>Wavelet-HHL_Kurtosis	The radiomics signatures extracted from DWI, ADC, T2WI, were combined to build a radiomics model. The top 30 features by mRMR were chosen	Radiomics model: LASSO regression, Rad-score. Clinical model: univariate analysis<br>Combined model: incorporating the significant clinical features with a binary logistics regression model to build a combined nomogram using a logistic regression algorithm.

Liqin Yang et al[29]	Manual delineation of the ROI on axial images of the T2WI, ADC and DWI by radiologist 1 (6 years of prostate MRI experience), reviewed by radiologist 2 (12 years of prostate MRI experience), for controversial lesions 2 radiologists more were consulted.<br><br>Two radiologist assed ECE by Mehralivand grade based on T2, ADC and DWI	Firstly, different target images of the same patient were matched spatially by Elastix software package (v. 4.10, http://elastix.isi.uu.nl/index.php). All scans were then interpolated to 1 x 1 x 1 cm3 to normalize the voxel spacing	Total of 2553 radiomics features: 42 morphology features, 54 first-order statistics features,<br>15 NGLDM, 42 GLCM, 48 GLRLM, 48 GLSZM, 72 GLCM, 2232 WTF	20 radiomics features were selected<br>9 ADC: GLCM,GLRLM,4 first order, 2GLSZM<br>6 DWI: GLDM, 6 GLSZM<br>5 T2WI: GLSZM , 2 first order,GLRLM,GLCM	Z-score method was applied to normalize the extracted features.<br>Near-zero variance, percentage of distinct values out of the number of total samples below 10%.<br>Pearson's correlation < 0.9.<br>Redundant features remotion based on recursive feature elimination (RFE)	Five classifiers were used to on the selected features to build the model<br>-LASSO (least absolute shrinkage and selection operator)<br>- DT (decision tree)<br>-RF (random forest)<br>- LR (logistic regression)<br>- SVM (support vector machine)<br><br>LASSO had the best results based on T2WI
Yang liu[27]	Manual delineation of the ROI by 2 radiologist	no	Total of 837 radiomics features: GLDM, GLCM, 48 GLRLM, GLSZM and wavelet	5 potential predictions (rad score):<br>2GLCM, 2 first order 1 GLRLM, more Gleason score (independent predictor)<br>P504s and age were not significant predictors.	student t and Mann-Whitney U test, was adopted to evaluate the significant features correlated with invasion	-LASSO (least absolute shrinkage and selection operator) and 10-fold-cross validation

Table S1: ITR (Intratumoral Region);PTR (Peri-Tumoral Ring);ROI (Region of Interest); VOI (Volume of Interest); ICC (Inter-reader Concordance Coefficient); T2WI(Weighted Image);ADC (Apparent Diffusion Coefficient); DWI(Diffusion-Weighted Imaging);
LBP3D (Local Binary Pattern in 3D); IF (Imaging Feature);
GLCM (Gray-Level Co-ocurrence Matrix), GLRLM (Gray-Level Run Length Matrix), GLSZM (Gray-Level Size Zone Matrix); NGTDM (Neighbouring Gray Tone Difference Matrix), GLDM (grey-level difference method); mRMR (minimum Redundancy Maximum Relevance); SRHGE: Short Run High Gray Level Emphasis
GNL (Gray-Level Nonuniformity); LRE (Long Run Emphasis); SZLGE (Small Zone Low Gray-level Emphasis); ZSN (Zone-Size Nonuniformity); SZE (Small Zone Emphasis); SZHGE (Small Zone High Gray-Level Emphasis); HGZE (High Gray-Level Zone Emphasis)
LHL GLSZ GLV(Gray-Level Size Zone, Grey Level variance); HLH GLRL (HighLowHigh Gray-Level Run Length Level Run Emphasis), HHH_glrlLRGLE (HighLowLow Gray-Level Run Length Level Long Run Low Gray Level Emphasis)
